# Treatment of cervical cancer with electronic brachytherapy

**DOI:** 10.1002/acm2.12657

**Published:** 2019-06-11

**Authors:** Sergio Lozares-Cordero, José Antonio Font-Gómez, Almudena Gandía‐Martínez, Anabela Miranda‐Burgos, Agustina Méndez‐Villamón, David Villa‐Gazulla, Verónica Alba‐Escorihuela, Sara Jiménez‐Puertas, Víctor González‐Pérez

**Affiliations:** ^1^ Department of Physics and Radiation Protection Miguel Servet University Hospital Zaragoza Spain; ^2^ Department of Radiation Oncology Miguel Servet University Hospital Zaragoza Spain; ^3^ Department of Physics and Radiation Protection Fundación IVO Valencia Spain

**Keywords:** cervical cancer, electronic brachytherapy, HDR, image‐guided brachytherapy

## Abstract

**Purpose:**

We report the first cervical cancer cases treated with interstitial electronic brachytherapy (eBT) at our hospital and compare them with plans made with high‐dose‐rate interstitial brachytherapy based on Ir192 (HDR‐BT).

**Materials and methods:**

Eight patients with cervical cancer were treated with the Axxent eBT device (Xoft, Inc.). Planning was with magnetic resonance imaging and computed tomography following the recommendations of the EMBRACE protocol. The dosimetry parameters of organs at risk (OAR) were evaluated for the bladder, rectum, and sigmoid colon (D2cc, D1cc, and D0.1cc). In addition, the V150 and V200 of irradiated tissue were compared for both eBT and HDR‐BT. All patients received intensity‐modulated external beam radiation therapy with a regimen of 23 sessions of 2 Gy followed by four sessions of 7 Gy of eBT performed over 2 weeks (two sessions followed by another two sessions a week later) following the EMBRACE recommendations. Each of the eight patients was followed to assess acute toxicity associated with treatment.

**Results:**

The doses reaching OAR for eBT plans were lower than for HDR‐BT plans. As for acute toxicity associated with eBT, very few cases of mucositis were detected. No cases of rectal toxicity and one case with grade 1 urinary toxicity were detected. The results at 1 month are equally good, and no relapses have occurred to date.

**Conclusions:**

The first results of treatment with the Axxent eBT device are promising, as no recurrences have been observed and toxicity is very low. eBT is a good alternative for treating cervical cancer in centers without access to conventional HDR.

## 1 INTRODUCTION

Cervical cancer is the second most frequent type of cancer in women, with a mean age at onset of 45 yr worldwide. It is also the most frequent type of cancer in developing countries, with more than 500,000 new cases diagnosed every year.^1^

The initial approach in patients with stage IA1‐IA2 cervical cancer is generally surgery, if possible, and radiotherapy or brachytherapy (BT) if not. Patients with advanced cancer (IB2‐IIA bulky‐IIB‐III‐IVA) receive chemotherapy and radiotherapy concomitantly, as well as BT, irrespective of whether they can undergo surgery.^2^

The use of BT as a key component in the treatment of cervical cancer is the main prognostic factor in local control of the disease.^3^

As with cancer at other sites, the response of gynecological cancer to radiation is dose‐dependent, with improved local control at higher doses.^4^ Better knowledge of tumor extension at diagnosis and the response to the treatment received has led to more realistic local treatment of cervical carcinoma, with adapted BT that is tailored depending on the findings at different points during the course of the disease. Therefore, BT has proven to be efficacious as a component of radiation therapy. The dose released by BT in contact with the tumor (4 fractions in 2 weeks) is at least equivalent to the dose administered during external beam radiation therapy (EBRT) on the pelvis for 5 or 6 weeks.^5^

Any nonsurgical treatment of gynecologic cancer with curative intent should combine EBRT and BT.^6^ The traditional approach has been low‐dose rate BT.^7^ For the last few years, the type of BT applied has been high‐dose rate BT (HDR‐BT) with Ir192,^8^ which makes it possible to optimize both the dose and patient comfort, since it can be administered on an outpatient basis, even though more sessions are necessary.

Technical advances in imaging and dosimetry have led to the use of computed tomography (CT) and magnetic resonance imaging (MRI) to locate the tumor and organs at risk (OAR) and to plan treatment based on real anatomy.^9^

A working group from GEC‐ESTRO published recommendations on contouring the target tumor and OAR, as well as on the dose volume parameters to be reported for image‐guided BT in definitive radiotherapy for locally advanced cervical cancer.^10^ The major advantage of this technique is the possibility of conforming the dose to both volume (3D) and time (4D). Thus, repetitive imaging performed before each BT implant makes it possible to adapt the BT dose to the anatomy of the individual patient, taking into account not only the position of the OAR but also tumor regression, which is often achieved with previous EBRT and chemotherapy.

The EMBRACE study,^11^ which was developed some time after publication of the GEC‐ESTRO recommendations, attempted to unify all of the processes involved in the management of cervical cancer by providing guidelines for the participating centers. A key feature is the use of MRI to better define the tumor and the OAR volumes. It allows to prescribe higher doses to the region of interest thus increasing the tumor control as shown in the Retro EMBRACE study.^12^ All of the participating centers used HDR‐BT with Ir192 in their treatments.

Electronic brachytherapy (eBT) has been evolving since the start of the 21st century^13^ and has become a treatment option for various tumor sites in different settings.^14^, ^15^, ^16^, ^17^, ^18^

The Axxent eBT unit (Xoft, Inc., subsidiary of iCAD, San José, CA, USA) provides treatment to patients with a miniature 50‐kVp x‐ray source that irradiates the tumor directly in skin cancer and with different applicators in the case of breast or gynecologic cancer.

The unit is constructed of a disposable mini‐x‐ray tube inserted into a flexible plastic sheath approximately 5.5 mm in diameter. Water is pumped around the source to reduce heating from the target. The electrons (20–50 keV) are emitted from a filament nearly 1 cm from the anode. The anode is made mainly of a microlayer of tungsten on an yttrium substrate that acts as a buffering layer and a small amount of silver that acts as a constituent of the brazing alloy. The anode transitions are from a spherical tip to a conical‐hemisphere section and finally to a cylinder. The source is mounted on a mechanical robot arm that is attached to a movable treatment control console.^19^

The unit can be used to treat nonmelanoma skin tumors, intraoperative radiotherapy in breast cancer, and postoperative treatment of endometrial and cervical cancer.

The case of skin tumors is problematic. High doses can reach bone (high atomic number), owing to the predominant photoelectric effect at low energies.^20^ Such a situation could arise in cases of cervical cancer, although, to date, we have had no reports of this problem at our center.

**Figure 1:**
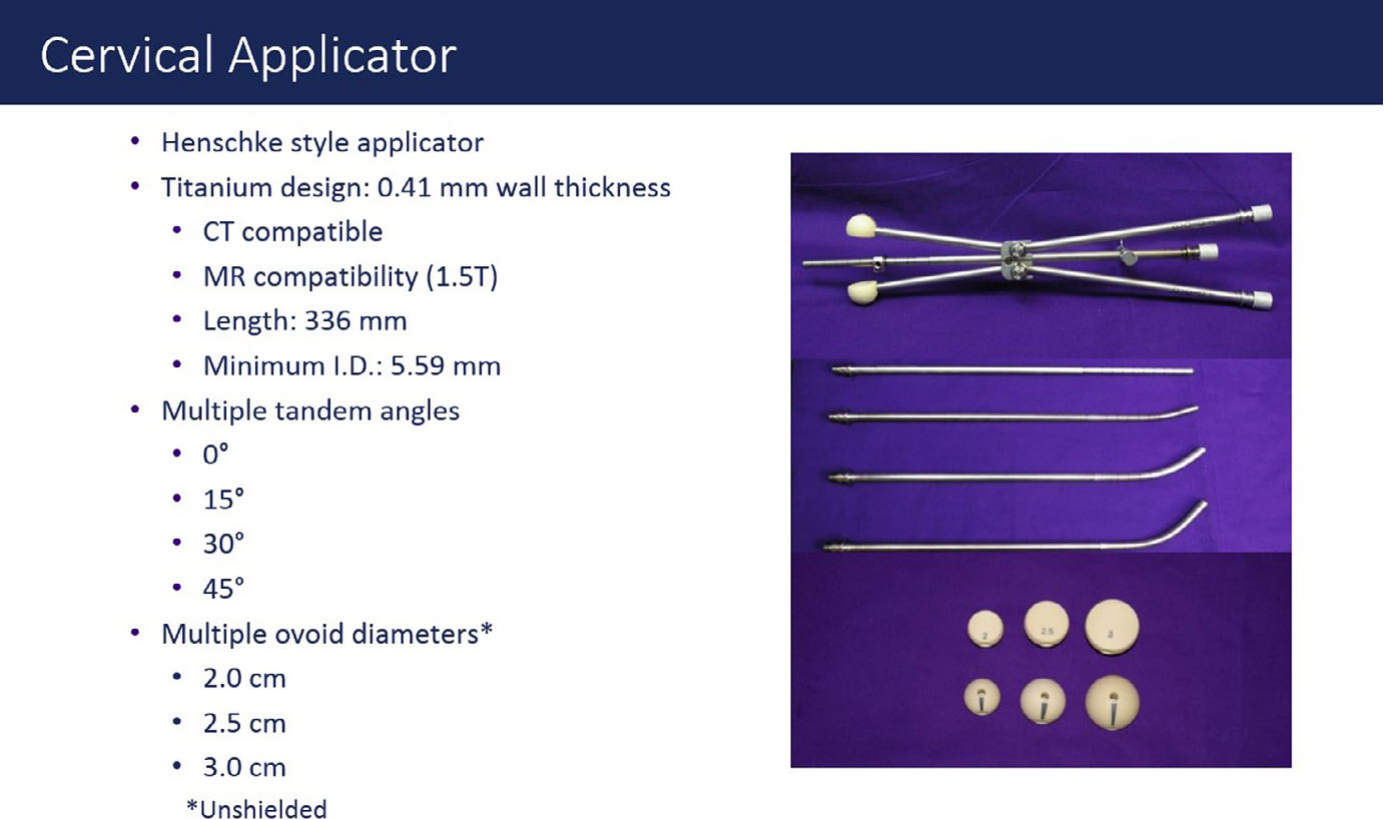
Cervical applicator.

It can also be used to treat cervical cancer in protocols that require treatment with HDR‐BT after chemotherapy and EBRT with a dedicated applicator (Fig. 1).

The eBT system at our center was acquired in May 2015 for treatment of skin cancer and intraoperative radiotherapy for breast cancer after tumor removal.

Postoperative treatment of endometrial cancer with the Axxent eBT unit was started in 2015, and in May 2016, we treated the first case of cervical cancer.

This therapeutic approach was traditionally applied in many centers throughout the world with HDR devices based on Iridium‐192 (Ir192, half‐life of 73.8 days and mean energy of 0.355 MeV) and different types of applicator, of which Fletcher applicators were the most common. In the current century, others have appeared and may help in treatment based on interstitial needles placed in various configurations.^21^

Retrospective studies on patients treated with Ir192 have compared endometrial cancer,^22^, ^23^ cervical cancer,^24^ and breast cancer.^25^ A lower dose was always found in the OAR in patients whose planning was based on eBT, although the patients in those studies were treated with Ir192. Another study comparing patients with endometrial cancer treated with eBT has reported favorable preliminary results.^26^

The objective of treatmenting with eBT is to provide an alternative with a portable device, thus conferring the advantage of mobility and obviating the need for a shielded room: with the energy used by the device, screening equivalent to 0.5 mm of lead on walls and doors is sufficient. Transport is no longer a factor since it does not involve radioactive sources. In addition, the device facilitates quality control and preparation and implementation of the treatment.^27^

## 2 MATERIALS AND METHODS

At our center, patients diagnosed with cervical cancer who are candidates for eBT after EBRT are selected from among those who, at their first evaluation for radiotherapy, are expected to have a residual tumor ≤3 cm with no parametrial involvement after EBRT. They are re‐evaluated after EBRT to determine whether the diagnostic parameters remain unchanged (see above).

In this study eight patients were treated using the Axxent (Xoft) device between May 2016 and June 2018 with the Axxent cervical applicator (Modified Henschke HDR Cervix Applicator‐Fig. 1), intrauterine probes angled at 15º, and ovoids of 2–3 cm. After excluding quality control time, each eBT source has a useful average clinical life of 750 min. If a source is not stable, it is withdrawn and replaced (Table 1). Each session lasted between 15 and 25 min.

**Table 1:** Characteristics of electronic brachytherapy sources.

Working life of eBT sources	750 minutes of clinical use
Deviation allowed with respect to calibration certificate	<10%
Deviation allowed with respect to first measure	<5%
Maximum energy	50 kVp
Average energy	29.6 keV
Treatment time for cervical treatment	15–25 min

Mean age was 59.8 yr (27–70 yr), with different tumor stages (Table 2). The eight patients received chemotherapy without surgery and adjuvant EBRT in the form of intensity modulated radiation therapy (IMRT) with a regimen comprising 23 sessions of 2 Gy each all over the pelvis, followed by eBT (28 Gy in four sessions). The sessions were administered twice weekly (Thursday and Friday) after radiation therapy. Planning was with MRI and CT scans acquired before the first session. The patient was then admitted for the second session the following day, after a new CT check‐up. The procedure is repeated the following week for the third and fourth sessions, with new MRI and CT images. OAR and target volumes are contoured in accordance with the EMBRACE protocol. The treatment planning system (TPS) used is Brachyvision‐Eclipse (Varian Medical System Inc., Palo Alto, CA, USA). The mean time from initiation of IMRT to the end of eBT was 52.5 days (49–58 days), and the time between the end of IMRT and the end of eBT was 10.6 days (7–15 days) (Table 2). The calculations are compared retrospectively with those for Ir192 sources and for each of the eight patients, with two plans per patient, giving a total of 32 different plans (16 for eBT and 16 for Ir192).

**Table 2:** Patients and treatments characteristics.

Patients	8
Age + Range (years)	59.7 (27–72)
FIGO Stage	%
IB1	12.5% (1)
IB2	12.5% (1)
IIA2	25% (2)
IIB	37.5% (3)
IIIB	12.5% (1)
Uterine probe inclination	15º (8)
Ovoids 3 cm	50% (4)
Ovoids 2.5 cm	25% (2)
Ovoids 2 cm	25% (2)
Time IMRT to eBT	10.6 (7–15) days
OTT	52.5 (49–58) days
Treatment time in tandem per fraction	14.6 (8.8–19.5) min
Treatment time in ovoids per fraction	3.5 (0.8–7.5) min

Proportions are given with number of total and median range.

FIGO stage is given in percentage of the total number of patients.

Time IMRT to eBT: time between the end IMRT treatment and the start of eBT.

OTT, Overall treatment time.

Planning was performed after a CT study (3‐mm slice thickness) and T2‐weighted MRI study (5‐mm slice thickness). Both sets of images were registered.

CT images are used to reconstruct the applicators appropriately; MRI is used to contour the target volumes and the OAR.

The high‐risk clinical target volume (HR‐CTV) and the intermediate‐risk clinical target volume (IR‐CTV) are contoured, and the bladder, rectum, and sigmoid colon as OAR following the recommendations of GEC‐ESTRO.^28^

After the medical physicist reconstructs the applicators and prepares the plan according to the dosimetric requirements of the EMBRACE protocol^10^ (Tables 3 and 4), the radiation oncologist approves it, and if appropriate, the patient is treated. Subsequently, an additional plan is drawn up for treatment with Ir192 source in order to compare both plans. The processing unit defined in the TPS for this purpose is Gammamed Plus with GammaMed Plus HDR source 0.9 mm (Varian Medical System Inc., Palo Alto, CA, USA).

**Table 3:** EMBRACE protocol dosimetric requirements based on ESTRO‐ABS recommendations for CTVs and OARs in EQD2.

Target	D90 HR‐CTV EQD2_10_	D98 HR‐CTV EQD2_10_	D98 IR‐CTV EQD2_10_
planning aims	>90 Gy < 95 Gy	>75 Gy	>60 Gy
Limits for prescribed dose	>85 Gy		
OAR	Bladder D_2cc_ EQD2_3_	Rectum D_2cc_ EQD2_3_	Sigmoid D_2cc_ EQD2_3_
Planning aims	<80 Gy	<65 Gy	<70 Gy
Limits for prescribed dose	<90 Gy	<75 Gy	<75 Gy

**Table 4:** EMBRACE protocol dosimetric requirements for BT (4 fractions of 7 Gy) after 46 Gy of EBRT in EQD2, %PD and dose per BT fraction.

D90 HR‐CTV		D98 HR‐CTV	
%PD	1 fraction	%PD	1 fraction
>107%	>7.5 Gy	>80%	>5.6 Gy
<117%	<8.2 Gy		
D98 IR‐CTV			
%PD	1 fraction		
>46%	>3.2 Gy		
Bladder: D2cc ≤ 90 Gy, D0.1cc ≤ 110‐115 Gy Bladder: D2cc ≤ 75–87%, D0.1 cc ≤ 107–113% Bladder: D2cc ≤ 5.3–6.1 Gy, D0.1cc ≤ 7.5–7.9 Gy			
Rectum: D2cc ≤ 75 Gy, D0.1 cc ≤ 80–85 Gy Rectum: D2cc ≤ 51–67%, D0.1 cc ≤ 75–81% Rectum: D2cc ≤ 3.6–4.7 Gy, D0.1 cc ≤ 5.3–5.7 Gy			
Sigmoid: D2cc ≤ 75 Gy, D0.1 cc ≤ 80–85 Gy Sigmoid: D2cc ≤ 60–67%, D0.1 cc ≤ 75–81% Sigmoid: D2cc ≤ 4.2–4.7 Gy, D0.1 cc ≤ 5.3–5.7 Gy			

Prescribed dose of BT treatment: 4 fractions of 7 Gy.

EQD2_n_, Dosage equivalent to 2 Gy per session for α/β = n according to the quadratic linear model. The limit value is the result of adding EBRT + BT; HR‐CTV, High‐risk clinical target volume; IR‐CTV, Intermediate‐risk clinical target volume; %PD, Percentage of prescribed dose.

The calculation algorithm is TG‐43, with specific parameters for the Axxent device.^29^

As for HR‐CTV and IR‐CTV, the values of the dose were determined to 90% (D90) and 98% (D98). HR‐CTV values are the same in the two treatment plans in each case, since the same normalization is applied throughout planning. We also determined V150 and V200, both of the HR‐CTV and of total tissue (sum of tumor tissue and healthy tissue), which receive 150% and 200% of the prescribed dose.

As for the OAR, the parameters are the same as those recommended in the EMBRACE protocol, and they are determined for all OAR of interest in the study, namely, bladder, rectum, and sigmoid colon. The parameters compared are D2cc (maximum dose, 2 cc), D1cc, and D0.1cc; toxicity is evaluated according to the parameters of the Radiation Therapy Oncology Group (RTOG).^30^

## 3 RESULTS

**Figure 2:**
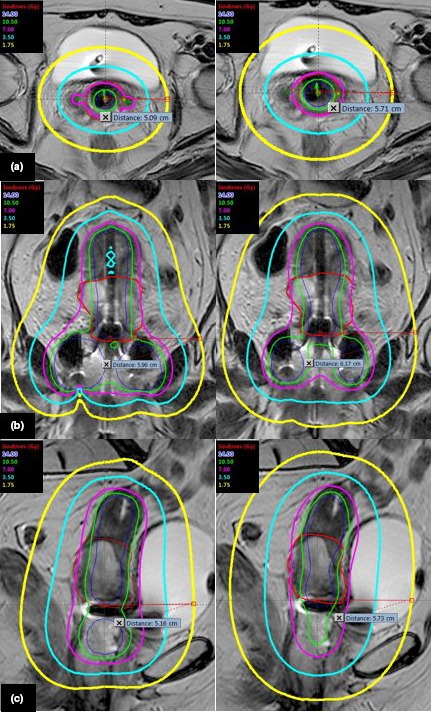
Isodose lines in Axial, Coronal, and Sagittal views for eBT and Ir192. (a) Axial view: Distances to 25% of the prescribed dose: 5.1 cm (eBT) and 5.7 cm (Ir192). (b) Coronal view: Distances to 25% of the prescribed dose: 5.9 cm (eBT) cm and 6.2 cm (Ir192). (c) Sagittal view: Distances to 25% of the prescribed dose: 5.2 cm (eBT) cm and 5.7 cm (Ir192). Red line: HR‐CTV contour. Prescribed Dose: 7 Gy per fraction.

Patients were followed up for a median of 13.4 months (2–28 months). Patients planned with eBT received a lower dose in the OARs than those planned with Ir192. The differences in the energy of both irradiation methods constitute the isodose lines that lead to the dose differences in the OARs (Fig. 2).

In the bladder, the average dose parameters for all the treatments compared (eBT vs Ir192) were 63% of the prescribed dose vs 66% for D2cc, 70% vs 73% for D1cc, and 84% vs 86% for D0.1cc. The differences were more remarkable in the rectum (30% vs 37% for D2cc, 32.9% vs 36% for D1cc, and 50% vs 56% for D0.1cc) and in the sigmoid colon (D2cc, 54% vs 57%; D1cc, 63% vs 66%; and D0.1cc, 86% vs 89%) with an average HR‐CTV volume of 16.6 cc (Table 5).

**Table 5:** Comparison of dosimetric parameters for Axxent eBx and Ir‐192 HDR.

EBRT (IMRT)	
Dose per fraction:	2 Gy
No fractions	23
Total dose:	46 Gy
BT (eBx or Ir‐192)	
Dose per fraction:	7 Gy
No fractions	4
Total dose:	28 Gy

Vol HR‐CTV: 16.6cc (10.1–38.7)		Dose per fraction (Gy)									
		Axxent‐eBx					Ir‐192				
		Mean (Gy)	SD (Gy)	%PD	Range (Gy)	Range(%PD)	Mean (Gy)	SD (Gy)	%PD	Range (Gy)	Range(%PD)
Target											
HR‐CTV	D98	7	0.7	100%	6.3–7.8	89–111%	7.1	0.8	101%	6.3–8.3	89–119%
	D90	8.5	0.9	121%	7.6–10.0	108–142%	8.4	0.8	120%	7.6–10	108–143%
IR‐CTV	D98	4	0.9	57%	3.0–5.4	42–76%	4.1	0.8	59%	3.4–5.3	48–76%
	D90	4.9	1.0	70%	3.7–6.5	53–93%	5	0.9	71%	4.1–6.4	58–91%
Organs at risk											
Bladder	D2cc	4.4	0.8	63%	3.9–6.1	55–87%	4.6	0.8	66%	4.1–6.3	59–90%
	D1cc	4.9	0.8	70%	4.3–6.7	61–96%	5.1	0.8	73%	4.5–6.9	64–99%
	D0.1cc	5.9	0.9	84%	5.4–8.1	77–115%	6	0.9	86%	5.5–8.2	79–117%
Rectum	D2cc	2.1	0.7	30%	1.3–3.5	19–49%	2.6	0.7	37%	1.9–3.9	26–56%
	D1cc	2.5	0.8	36%	1.5–3.8	21–54%	3	0.8	43%	2.1–4.1	30–59%
	D0.1cc	3.5	1.0	50%	1.9–4.4	26–63%	3.9	1.0	56%	2.5–4.9	36–70%
Sigmoid	D2cc	3.8	0.7	54%	3.1–5.2	44–74%	4	0.7	57%	3.3–5.3	47–76%
	D1cc	4.4	0.9	63%	3.7–6.2	52–88%	4.6	0.9	66%	3.9–6.4	55–91%
	D0.1cc	6	1.4	86%	4.6–8.5	65–121%	6.2	1.4	89%	4.8–8.7	69–124%

D98, D90: Dose to the 98% or 90% of the HR‐CTV or IR‐CTV volume in Gy.

D2cc, D1cc, D0.1cc: Doses to the volume of 2 cc, 1 cc, or 0.1 cc in Gy and in %PD (percentage of prescribed dose).

SD, standard deviation; Vol HR‐CTV, Volume of HR‐CTV, average, and range; HR‐CTV, high‐risk clinical target volume; IR‐CTV, intermediate‐risk clinical target volume.

**Figure 3:**
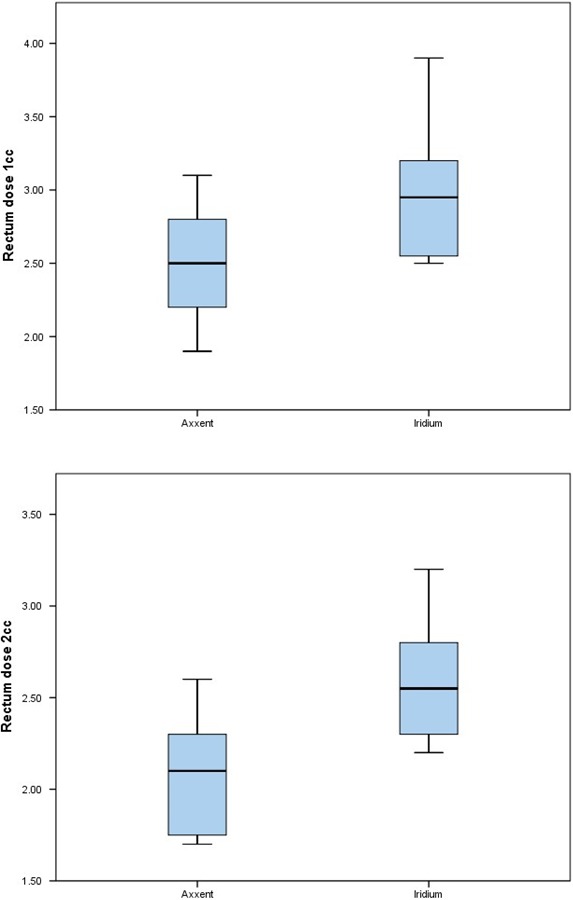
Box and Whisker Plot: D1cc and D2cc of rectum.

By applying these data and the t test, we see that the difference is significant for cases of D2cc and D1cc of the rectum (*P* < 0.05) (Fig. 3).

Although D90 and D98 for HR‐CTV were the values used to standardize the plans, we did observe a small difference in the coverage of IR‐CTV—57% of the prescribed dose for eBT vs 59% for HDR‐BT—although both were above the objective set by EMBRACE (>46%, Tables 3 and 4). In addition to the differences in the IR‐CTV coverage percentage, it is useful to observe the differences in dose equivalent to 2 Gy (EQD2), taking into account the contribution of EBRT and the part of eBT or Ir192 in each case (Table 6). V150 and V200 for HR‐CTV were greater for the cases calculated with Axxent than for those calculated with Ir192, although the difference was very small owing to the volume of the tumors treated. In addition, even though tissue volumes that received 150% and 200% of the dose prescribed were included (Table 7), given the low energy of eBT compared to Ir192 also observed an increase in the dose in the vaginal wall in contact with the surface of the ovoids of 13% on average, although no cases of acute mucositis were observed. Of the eight patients treated at our center, only one had grade 2 acute toxicity (RTOG) (Table 8). One month after treatment, vaginal toxicity had disappeared. Rectal toxicity and urinal toxicity were minimal (Table 8). To date, no patients have experienced recurrence of their disease.

**Table 6:** EQD2 for Axxent and Ir‐192. BT and EBRT (IMRT) obtained adding doses according to linear quadratic model.

		AXXENT				Ir‐192			
		EQD2(Gy)				EQD2(Gy)			
		BT	BT + IMRT	%PD	%SD	BT	BT + IMRT	%PD	%SD
α/β = 10Gy									
Target									
HR‐CTV	Prescription dose.	39.7	85.7	100%		39.7	85.7	100%	
	D98	39.7	85.7	100%	9%	40.5	86.5	101%	14%
	D90	52.4	98.4	121%	13%	51.5	97.5	120%	17%
IR‐CTV	D98	18.7	68.7	57%	13%	19.3	65.3	59%	8%
	D90	24.3	70.3	70%	14%	25.0	71.0	71%	10%
α/β = 3Gy									
Organs at risk									
Bladder	D2cc	26.6	72.6	64%	9%	28.0	74.0	66%	9%
	D1cc	30.0	76.0	69%	10%	33.0	79.0	73%	10%
	D0.1cc	41.4	87.4	84%	12%	43.2	89.2	86%	12%
Rectum	D2cc	6.1	52.1	23%	4%	11.6	57.6	37%	5%
	D1cc	9.0	55.0	31%	5%	14.4	60.4	43%	6%
	D0.1cc	14.6	60.6	43%	7%	21.5	67.5	56%	8%
Sigmoid	D2cc	19.8	65.8	53%	8%	22.4	68.4	57%	8%
	D1cc	23.8	69.8	59%	9%	28.0	74.0	66%	9%
	D0.1cc	38.1	84.1	79%	12%	45.6	91.6	89%	13%

α/β = 10 Gy for target and 3 Gy for OAR according to EMBRACE.

EQD2, Equivalent dose to 2 Gy per fraction in EBRT; %PD, Percentage of Prescribed Dose; %SD, Standard Deviation of %PD; IMRT, intensity modulated radiation therapy; BT, brachytherapy; HR‐CTV, high‐risk clinical target volume; IR‐CTV, intermediate‐risk clinical target volume.

**Table 7:** V150 and V200 for HR‐CTV and for soft tissue.

	Axxent‐eBx (%HR‐CTV Volume)			Ir‐192 (%HR‐CTV Volume)		
	Mean (%)	SD (%)	Range (%)	Mean (%)	SD (%)	Range (%)
V150	73%	9%	62–90%	73%	10%	57–91%
V200	51%	11%	36–71%	51%	12%	26–72%
Soft tissue 150%	132%	42%	95–246%	124%	26%	96–169%
Soft tissue 200%	67%	29%	34–153%	63%	12%	49–87%

Mean, standard deviation and range of V150, V200: Volume of HR‐CTV and Soft tissue with 150% and 200% of the prescribed dose in percentage of the HR‐CTV volume.

HR‐CTV, high‐risk clinical target volume.

**Table 8:** Acute and 1 month toxicity of patients.

	Grade 0	%	Grade 1	%	Grade 2	%
N = 8						
Acute vaginal mucositis	4	50%	3	37.5%	1	12.5%
Acute rectal toxicity	7	87.5%	1	12.5%	0	0%
Acute urinary toxicity	6	75%	2	25%	0	0%
Toxicity (1 month)						
Vaginal toxicity	7	87.5%	1	12.5%	0	0%
Rectal toxicity	8	100%	0	0%	0	0%
Urinary toxicity	8	100%	0	0%	0	0%

Grade 0,1,2: Int J Radiat Oncol Biol Phys. 1995 Mar 30;31(5):1341‐6. Toxicity criteria of the Radiation Therapy Oncology Group (RTOG) and the European Organization for Research and Treatment of Cancer (EORTC).

N, number of patients.

## 4 DISCUSSION

We obtained acceptable results after the first 28 months (mean follow‐up, 13.4; range, 2–28 months) using the Axxent eBT device for treatment of cervical cancer with EBRT and chemotherapy, thus indicating that this option is a good alternative in BT.

The dose prescribed in each eBT treatment was administered without taking into account the fact that the mean beam energy was much lower than in Ir192 (26 keV vs 355 keV) and, therefore, without taking into account the differences in the relative radiobiological effectiveness (RBE) expected for low‐energy radiation.^31^, ^32^

One clinical study showed that reducing the dose prescribed for treatment of nodular and superficial basal cell carcinoma using eBT and based on a different RBE reduced control of the tumor from 95% to 90%, thus demonstrating better control for the standard prescription.^33^

In our study, we did not modify treatment owing to differences in RBE with respect to Ir192 because of the good results achieved with treatment administered to the endometrium^26^ and the contradictory results reported in several published studies. Modification of the prescribed dose continues to be controversial in low‐energy cases.^34^

We can achieve the same coverage of the cervix as during planning when Ir192 is used in all of the cases presented and the dose to OAR can be reduced, even though V150 and V200 of the planning target volume increase very slightly. This could have led to an increase in the number of cases of acute mucositis in the study population; however, the toxicity results, together with the reduced dose administered to OAR, lead us to conclude that eBT is a good alternative to treatment with Ir192 in cases of cervical cancer.

In their study of 10 patients, Mobit et al.^24^ evaluated dosimetric differences and similarities between treatment plans generated with the eBT source of Axxent and Ir92 for tandem applicators and ovoids. The authors reported a difference between the two approaches (eBT vs Ir192) for D2cc in the bladder (43.9% vs 58.7% of the prescribed dose), in the rectum (54.9% vs 60.9%), and in the sigmoid colon (44.1% vs 56%). However, in this case, patients were treated with Ir192, and the eBT plans were calculated prospectively.

As for the calculation method used, it is recommended to consider tissue composition and to perform the calculations based on Monte Carlo models.^35^ Our calculations were based on TG‐43, which was modified for eBT,^29^, ^36^ with no correction for heterogeneity, since this was the algorithm used in our TPS and in many other hospitals, for which our results will prove useful.

The sample of patients selected only included tumors with no parametrial extension so as not to require insertion of interstitial needles during treatment. Since this modality is not yet available in the Axxent model, patients requiring interstitial needles were referred to another center.

Results for the healthy tissue volume that received high doses are slightly higher in the case of eBT, where the tissue irradiated with 150% of the prescribed dose corresponds to 132% of the HR‐CTV volume, compared with 124% in cases planned with Ir192. For tissue irradiated with 200%, these values are 67% vs 63% (Table 7). The differences do not represent an excessive volume in each individual case and, more importantly, do not correspond to an increase in the number of cases of mucositis.

The dose in OAR is slightly lower in the case of eBT, and cases of toxicity associated with doses in OAR are minimal (Table 8).

We did not encounter problems of overdosing in bone, because we used low energies.^20^ However, overdosing should be avoided in order to reduce the number of fractions and thus prevent this problem from arising. We believe that the scheme proposed by EMBRACE is adequate in this respect.

Both dosimetry requirements of the EMBRACE protocol were fulfilled for both types of treatment, although D0.1cc in the sigmoid colon would be above tolerance for Ir192. However, as stated above, this does not lead to more cases of mucositis. These results, together with the absence of relapses to date, allow us to be optimistic with respect to determining a good eBT‐based alternative to this treatment approach, although longer follow‐up is necessary before we can confirm our findings. In addition, we must await the findings of other groups.

## 5 CONCLUSION

Treatment with eBT represents a huge advantage in centers with no HDR device, although more clinical results, results for local control, and results based on a longer follow‐up are necessary. Our data are based on only eight patients, thus reducing the importance of the conclusion. Nevertheless, they are a promising beginning for treatment of cervical cancer with electronic brachytherapy. The doses in the OARs are lower with eBT, although the difference is not significant in the bladder or sigmoid colon. They are significant, however, for the D2cc and D1cc parameters in the rectum. The broad experience accumulated over the years with Ir192 treatments makes it the reference for cervical cancer, eBT could be an alternative in cases where treatment with Ir 192 is not available, as long as the results obtained continue to be satisfactory.

The eBT device is a useful addition in centers with an HDR device. Its mobility and versatility mean that it can be used as a complementary facility to treat cervical cancer and for intraoperative radiotherapy in breast cancer, in skin cancer, and in postoperative treatment of endometrial cancer.^26^

In areas with a high incidence of cervical cancer and few Ir192‐based HDR devices, eBT could be a good alternative for patients who live far from a major hospital. In addition, given that treatment does not require a bunker for administration and the device is easily transported, this option is much more economically viable than others.

## CONFLICT OF INTEREST

No conflict of interest.
